# Use of *Frankia* and Actinorhizal Plants for Degraded Lands Reclamation

**DOI:** 10.1155/2013/948258

**Published:** 2013-11-11

**Authors:** Nathalie Diagne, Karthikeyan Arumugam, Mariama Ngom, Mathish Nambiar-Veetil, Claudine Franche, Krishna Kumar Narayanan, Laurent Laplaze

**Affiliations:** ^1^Laboratoire Mixte International Adaptation des Plantes et Microorganismes Associés aux Stress Environnementaux (LAPSE), 1386 Dakar, Senegal; ^2^Laboratoire Commun de Microbiologie IRD/ISRA/UCAD, 1386 Dakar, Senegal; ^3^Institute of Forest Genetics and Tree Breeding, Forest Campus, R. S. Puram, Coimbatore 641 002, India; ^4^Département de Biologie Végétale, Université Cheikh Anta Diop (UCAD), 5005 Dakar, Senegal; ^5^Equipe Rhizogenèse, UMR DIADE, IRD, 911 Avenue Agropolis, 34394 Montpellier Cedex 5, France

## Abstract

Degraded lands are defined by soils that have lost primary productivity due to abiotic or biotic stresses. Among the abiotic stresses, drought, salinity, and heavy metals are the main threats in tropical areas. These stresses affect plant growth and reduce their productivity. Nitrogen-fixing plants such as actinorhizal species that are able to grow in poor and disturbed soils are widely planted for the reclamation of such degraded lands. It has been reported that association of soil microbes especially the nitrogen-fixing bacteria *Frankia* with these actinorhizal plants can mitigate the adverse effects of abiotic and biotic stresses. Inoculation of actinorhizal plants with *Frankia* significantly improves plant growth, biomass, shoot and root N content, and survival rate after transplanting in fields. However, the success of establishment of actinorhizal plantation in degraded sites depends upon the choice of effective strains of *Frankia*. Studies related to the beneficial role of *Frankia* on the establishment of actinorhizal plants in degraded soils are scarce. In this review, we describe some examples of the use of *Frankia* inoculation to improve actinorhizal plant performances in harsh conditions for reclamation of degraded lands.

## 1. Introduction

In recent years, land degradation has increased considerably due to climatic factors as well as human intervention resulting in a reduction in fertility, biodiversity, and productivity. Since tropical countries are characterized by rapid demographic growth, the population dependency on ecosystems is high resulting in ecosystem degradation. Thus, the rehabilitation of degraded lands is critical. To overcome the problem of the lack of fertility in degraded soils, fast growing nitrogen fixing trees such as actinorhizal plants in combination with biofertilizers are used [1, 2]. Actinorhizal plants are nitrogen-fixing plants from 8 families including *Betulaceae*, *Casuarinaceae*, *Coriariaceae*, *Datiscaceae*, *Elaeagnaceae*, *Myricaceae*, *Rhamnaceae*, and *Rosaceae*, [3, 4] distributed in 25 genera and approximately 200 angiosperms species. Generally, these nitrogen-fixing plants are able to grow in poor and disturbed soils and are well adapted to abiotic stresses such as drought, salinity, and flooding [5]. Among them, plants of *Casuarinaceae* and *Betulaceae *families are the most widely planted around the world for the rehabilitation of degraded lands [6]. In addition to the positive aspects of actinorhizal plants on the remediation of degraded ecosystem, some actinorhizal plants such as *Hippophae rhamnoides* (sea buckthorn), *Hippophae L*., and *Rubus ellipticus* (Yellow Himalayan raspberry) are used as food ingredients or for medicinal purposes [7–9]. Actinorhizal plants are also pioneer species and have a potential role to enhance plant establishment on disturbed sites, to improve soil fertility and stability. 

In degraded soils, one of the scarcest nutrients is nitrogen. The symbiotic relationship with nitrogen-fixing bacteria *Frankia* increases soil fertility and enhances the performance of trees during their plantation in degraded lands. Upon establishment, actinorhizal tree seedlings must be inoculated in nursery or in the field [10]. The success of establishment of *Casuarina* plantation in degraded sites depends on the effective nodulation by appropriate *Frankia* strains [2, 11]. However, studies related to the beneficial role of the nitrogen fixing actinorhizal bacteria on actinorhizal plants for the rehabilitation of degraded soils are very scarce. In this paper, we review these efforts and highlight the positive role of *Frankia* in improving actinorhizal plant performances in harsh conditions. 

## 2. *Frankia*

**Figure 1:**
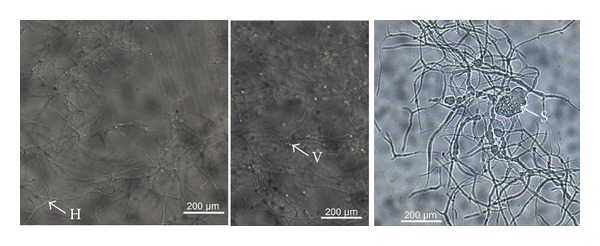
Structures of *Frankia* bacteria H: hyphae, V: vesicle, and S: sporangia.

*Frankia *is a gram-positive nitrogen-fixing actinobacterium that forms a symbiotic association with actinorhizal plants. It is a filamentous free-living bacterium [12] found in root nodules or in soil [13]. The genus* Frankia *has been classified in the order of Actinomycetales on the basis of morphology, cell chemistry, and 16S rRNA sequences [14]. Genomic studies have been undertaken to characterize *Frankia* [15–17] and to better understand the functioning of actinorhizal symbiosis. Among species of *Frankeniaceae*, the capacity to fix nitrogen is restricted to *Frankia* [3, 18]. This microaerophilic bacterium is characterized by a high GC% content and a slow growth rate [19]. In liquid culture and depending on the condition of culture, *Frankia* forms hyphae and multilocular sporangia which are located on hyphae either terminal or intercalary [20] (Figure 1). Ultrastructure showed that the hyphae, free living structures are septate, and sporangia are multilocular and contain the spores, the effective propagules of the bacteria. Vesicles are the site of nitrogenous activity and are generally formed when nitrogen is very limited in the medium [21] (Figure 1). Due to the presence of resistant structure in culture, *Frankia* inoculum is easier to conserve than *Rhizobium* inoculum [22]. However, *Frankia* morphology in the nodule varies according to host plant. The size of hyphae and the presence or the absence of vesicles depends on the bacterial partner [23, 24].

The symbiosis between actinorhizal plants and *Frankia* induces the formation of a perennial root organ called nodule, wherein bacteria is hosted and nitrogen is fixed [25, 26]. In the field, actinorhizal nodule can have variable forms and colours [27]. Comparison of actinorhizal and leguminous nodules shows that morphology, anatomy, origin, and functioning of nodules are different for these two nitrogen-fixing plants [28]. Two types of nodule formation occur in actinorhizal symbiosis: the intercellular and the extracellular infection [29].

## 3. Actinorhizal Plants

Actinorhizal plants are grouped in the clade of Rosid I [30]. With the exception of two species belonging to *Datiscaceae* family, actinorhizal plants are mostly trees or woody shrubs [4]. They are found on all continents with the exception of Antarctica [31]. *Casuarina* and *Alnus* are the most important and widely spread actinorhizal plants due to their uses in soil reclamation, agroforestry systems, dune stabilization, and windbreaks [32]. They are generally pioneer species that colonize disturbed environments with low soil fertility and facilitate the establishment and development of subsequent plant communities [33].

Among the actinorhizal plants, *Casuarinaceae* trees are widely distributed in tropical areas [34]. This family is composed of 4 genera with 96 species. These plants are originated from Australia, South-East Asia, Malaysia, Melanesian, and Polynesian regions of the Pacific, New Guinea [35, 36]. *Casuarina* species are able to grow well under a range of stresses such as drought, flooding, salinity, and sites polluted by heavy metal [27, 37]. *Casuarina* plantations improve physical and microbiological quality of degraded soils [38]. These plants have an important ecological and economical role in tropical countries. They contribute to the improvement of soil fertility by fixing nitrogen and producing thick leaf litter from needles that can be used as compost by farmers [2, 39–41].

**Figure 2:**
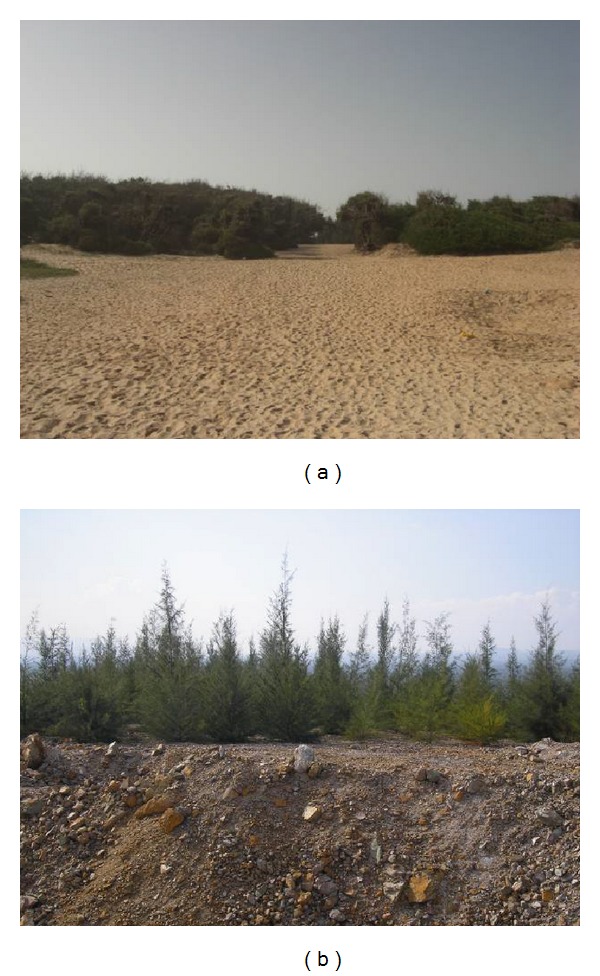
(a) *Casuarina equisetifolia* plantation in Niayes region for fixation of dunes, Senegal; (b) *C. equisetifolia* plantation in Magnesite mined out lands, India.

*Casuarina* trees are widely used for the rehabilitation of degraded lands in South Africa, Kenya [42]. They are used as windbreaks to protect adjacent crops and fix sand dunes in Senegal (Figure 2(a)), Tunisia, Egypt, China, and India [2, 36, 43]. In agroforestry system, these trees are used to improve soil fertility and increase crop yield (e.g, intercropping with legumes) in China and India [44]. *Casuarina* trees are also used in the production of smokeless fuelwood with a high calorific value, and hardwood in the construction of houses in Benin [45] and in the production of paper pulp wood in India [46]. In Asia, shelter belts formed by *Casuarina* trees have played a major protective role during typhoons and tsunami [44]. Association with *Frankia* increased *Casuarina* growth and biomass [2, 47, 48]. Furthermore, in this symbiotic relationship, bacteria confer to plants a high resistance to abiotic and biotic stresses [27, 49]. By using *C. equisetifolia* inoculated with *Frankia Ceq1*, Tani and Sasakawa [37] showed that actinorhizal symbiosis plays an important role in the reclamation of degraded lands. In India, about 5,000,000 ha are planted with *C. equisetifolia* (Figure 2(b)) and produce 10 million tonnes of pulpwood [50]. Recognising the importance of symbiotic association between *Casuarina*-*Frankia*, farmers have begun to use these biofertilizers through inoculation with crushed nodules [50]. The positive role of *Casuarina* and its ability to be propagated by seed, cutting, and tissue culture [44] makes it an ideal species for the reclamation of degraded lands. However, *C. equisetifolia* clones have been reported to show marked variation in their ability to tolerate salt stress [51–54]. Thus, identifying stress tolerant clones that can grow in degraded lands are crucial for the rehabilitation of these lands.

Like *Casuarina*, *Betulaceae* contains members that play an important role in improving soil fertility [55]. They are used in the production of firewood, pulp, and timber. These species are also used in land reclamation, agroforestry, and as windbreak to avoid erosion [56, 57] and promote the establishment of the more nutrient demanding plants [58]. Inoculation with *Frankia* increases the growth and biomass of *Alnus* [59, 60]. Furthermore, alder growth is higher when plants are inoculated with more *Frankia* strains [57]. Inoculation with *Frankia*, increases the leaf N content of *Alnus glutinosa* [61]. Alder plants inoculated with *Frankia* have a higher productivity, a higher shoot length, root length, dry matter production, and chlorophyll content [62]. Under nursery conditions, *Frankia* provides good alder plants to be used in reforestation programs [60]. Inoculation with *Frankia *increases the survival rate of transplanted seedlings in the field [63]. A plantation of *Alnus acumita* increases soil N content by about 279 kg h^−1^ [64]. Some other species of actinorhizal plants are also used as pioneer plants such as *Coriaria* and *Datisca *[65]. 

When compared to legume-rhizobia symbiosis, actinorhizal symbiosis fixes at similar high rate of N_2_ estimated at around 240–350 kg ha^−1^ y^−1^ [3].

## 4. The Nitrogen Fixing Bacteria *Frankia* Improves Actinorhizal Plant Performance in Reforestation Programs

### 4.1. Actinorhizal Symbiosis and Plant Growth

Inoculation with the nitrogen-fixing bacteria *Frankia* improves the nutrient status and enhances actinorhizal plant development [2, 59, 66, 67]. However, the successful establishment and growth of actinorhizal plants in nutrient deficient and/or marginal soils depends on the formation of the symbiotic relationship between plant and the nitrogen-fixing bacteria. Thus, to optimize association between the actinorhizal/*Frankia,* an efficient combination in nitrogen fixation well adapted to environmental conditions is recommended [2].

In fields, inoculation with* Frankia* is commonly carried out with crushed nodule,* Frankia* suspension, *Frankia *enrobed in alginate bead, soil containing *Frankia*, or leaf litter from around nodulated plants [10, 39]. The response to *Frankia* inoculation is strongly linked to factors such as provenance source, *Frankia* strain, and nutrients status of the site such as nitrogen [68]. *Frankia* inoculation in nursery and field conditions is beneficial to *Casuarina* given that it reduces transplantation shock [22, 68]. Another study [69] showed that Casuarinas generally do not form nodule outside their zone of origin; however, nodulation occurs when they are inoculated with* Frankia* from the plants' zone of origin. When *Casuarina* trees are planted in sites where they have not previously been planted, inoculation with *Frankia* is recommended for the successful establishment of the plantation [44] given that *Casuarina* strains are generally absent in zone without the host plant [69]. However, for some actinorhizal species infective *Frankia* strains can be found in zone without the host plant [70, 71]. Generally, arid soils are not infected with *Frankia* [71] but plant bioassays have demonstrated that members of the *Frankia *survive and remain infective in soils that are devoid of host plants [72]. 

Therefore, it becomes important to transfer stress tolerant actinorhizal plants along with effective *Frankia* strains during reforestation programs. Attention also needs to be given to match *Frankia *strains, plant, and environment, as the response of different clones to nodulation by *Frankia* may differ in different environments [2]. 

*Casuarina* plantations are spread all over Senegal and used for multiple purposes (Figure 2). In the coastal sandy dunes of the Niayes region of Senegal, *C. equisetifolia* was established in 1925 to fix dunes (Figure 2(a)) and to protect adjacent crops. Presently, about 10,000 ha are planted with *C. equisetifolia* along this coastal zone [74]. Inoculation with *Frankia* was achieved in the Niayes region later in 1976 during a reforestation program with support from FAO [75]. Based on Maggia's studies [76], nodules have been found in *C. equisetifolia* trees planted before 1976. This nodulation may be due to the natural presence of *Frankia* strains in Senegal or to the presence of *Frankia* associated with Casuarina plant from Australia and established in Senegal in 1925. To inoculate *Casuarina* plants in the Niayes region, the nodules from the *Casuarina* plantation established before were crushed and soaked for 4-5 days in water and used for irrigating plants [43]. About two weeks after inoculation, nodulation was observed in root system of inoculated plants [77]. In the Niayes region, association of *Frankia* with *Casuarina* plants enhanced plant growth and development [78], improved soil fertility, enhanced vegetable production largely used by the local population, and increased population incomes [43].

**Figure 3:**
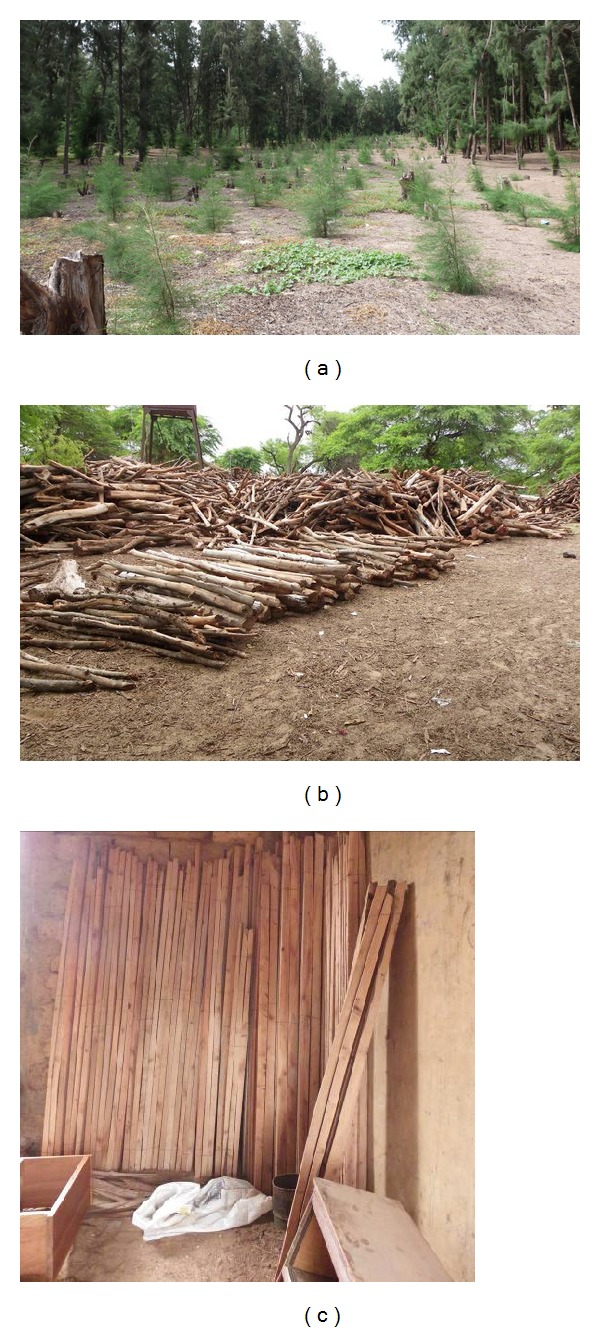
*C. equisetifolia* plantation in Mboro: (a) old trees are replaced by young seedlings; (b) and (c) uses of *C. equisetifolia* wood as firewood (b) or timber (c).

Pure cultures of *Frankia* were used to inoculate *Casuarina* plantations in 1984 in the Notto region of Senegal [39]. Plants were inoculated in a nursery condition before transplantation with *Frankia *strains *ORS 021001*. A few years after transplantation on fields, results obtained showed that *Frankia* strains were very effectively associated with *C. equisetifolia* [39]. Actinorhizal plants inoculated with *Frankia* were taller (Table 1) and had better photosynthesis activity, a higher shoot biomass, and a higher N_2_ content in roots and needles (Table 2). *Casuarina* plantations are replenished in Senegal by planting young seedlings inside the old *Casuarina* plantations (Figure 3(a)) to improve soil fertility and increase agricultural yield and wood production.

Plants inoculated with *Frankia* are also used in China for the rehabilitation of degraded lands. In 2001, more than 6 species of *Casuarina* were planted in degraded lands and inoculated with *Frankia*. Results from these experiments showed that *Frankia* application improves survival, biomass productivity, and plant growth [44].

For other actinorhizal species, such as members of *Betulaceae*, inoculation with *Frankia* has shown similar positive effects by improving plant growth and development in marginal soils. *Alnus* plants inoculated with *Frankia* have a higher productivity, higher shoot length, root length, dry matter production, chlorophyll content, and leaf N content [59, 61, 62]. Studies carried out by Lefrançois et al. [79, 80], showed that *Frankia*-alder symbiotic relationship improves soil quality. Taken together, inoculation of actinorhizal plants with *Frankia* is a promising tool for the rehabilitation of degraded lands.

### 4.2. Actinorhizal Symbiosis and Environment Stresses

Actinorhizal plants are generally tolerant of abiotic stresses. This tolerance can be improved when plants are associated with *Frankia* [81]. The actinorhizal plant-*Frankia *system is widely used for reclaiming lands affected by abiotic stresses [82]. Most of the *Frankia* strains were resistant to an elevated level of several heavy metals [83] and also to salinity [84]. In association with actinorhizal plants, *Frankia* strains resistant to abiotic stresses confer and/or increase capacity of the plant partner to tolerate abiotic stresses. Generally *Frankia* strains are absent or rare in stressed soils, which indicate the requirement of inoculation of the host plant before transplantation for rehabilitation of saline soil [85]. 

Some *Frankia* strains are very tolerant to salinity and can be used as biofertilizers in land affected by salt [84]. It has been demonstrated that nodulation occurred under saline conditions until 300 mM, approximately 28 dSm^−1^ [37]. Given that *Frankia* improves plant performance in stressed conditions [37], the symbiotic association between *C. equisetifolia* and *Frankia *can be widely used for the recovery of saline soils. However, selection of compatible salt tolerant *Frankia* and host plant is a key for obtaining the right actinorhizal plant-*Frankia* for rehabilitation of salt affected soils [37, 86] since differences in salt tolerance between individual* Frankia* strains have been shown by Ngom et al. (unpublished).

Actinorhizal symbioses are a biological tool generally used for the remediation and revegetation of soils affected by salt, heavy metal, oil, and so forth [62, 87]. It has been demonstrated that alder-*Frankia* symbiosis improves remediation capabilities and enhances soil quality by improving soil nutrients, pH, and cation exchange capacity and enhancing plant performance in these harsh conditions [80]. To restore the landscape of the Bamburi Cement Factory in Mombasa, Kenya, R. D. Haller initiated some plantation by testing 26 species in 1971. After six months, only three species, *C. equisetifolia *sp., *Conocarpus lancifolius sp*., and coconut palm survived. Among them, *C. equisetifolia* J.R et G. Forst was the most adapted to this environment where it acts as pioneer plant. In this reclamation program, Casuarina plants inoculated with *Frankia* showed a higher survival rate after transplantation [88].

Inoculation with *Frankia* has a beneficial effect on restoration and reforestation of bauxite mine spoils [50]. Their studies showed that in bauxite mine spoils the growth and nutrients uptake (N, P, K) of plants inoculated with *Frankia* was higher than those of noninoculated plants (Table 2). Beside, the important role of *Casuarina* on lands reclamation, the symbiotic relationship between *Frankia*-actinorhizal plant can also be used as a biocontrol tool against diseases such as bacterial wilt (*Ralstonia*), cataplexy (*Rhizoctonia* sp.), powdery mildew (Oidium *sp.*), Hexenbesen (mycoplasma-like organism; bacteria-like organism), and canker (*Phomopsis sp*.) [89]. Given that actinobacterium is a group of bacteria that generally produces antagonistic compounds against pathogens [90], the establishment of actinorhizal symbiosis could mitigate the development of plant disease [91]. In nursery experiments, results obtained by kang [92] showed that *Casuarina* plants inoculated with *Frankia *are more resistant to bacterial wilt disease than noninoculated plants. Furthermore, it has been demonstrated by [49] that *Frankia* strains could counterbalance root rot of *C. equisetifolia* caused by *Rhizoctonia *sp. There is a positive correlation between the dose of *Frankia*, the efficiency of disease control, and also nodule formation. Taken together, these results highlight the positive role of *Frankia* as an eco-friendly tool for the control of plant disease and the improvement of agricultural productivity [1].

## 5. Conclusion

Ecosystem degradation leads to soil infertility and crop losses therefore leading to decreased food security. Plantation of pioneer trees such as actinorhizal plants that are well adapted to such harsh conditions is a promising tool for restoring these degraded lands. However, for the successful establishment of the plantation, care should be taken to select appropriate tree species for a specific environment. In the context of the significant intraspecies variation in their tolerance to salinity, it becomes important to select tolerant clones for plantation programmes. To reduce the transplantation shock during reforestation programs and to increase the productivity of these plantations, *Frankia* inoculation must be carried out during the nursery stage. Association between actinorhizal plant and *Frankia* must be optimized by selecting a more efficient symbiosis. Thus, appropriate combinations of actinorhizal plants as well as compatible *Frankia* strains well adapted to environmental conditions are recommended. Given that the symbiotic microorganism* Frankia* significantly improves performance of actinorhizal plantations, it becomes very important to enhance *Frankia* production particularly in arid and semiarid areas for large-scale adoption by farmers.

## Figures and Tables

**Table 1:** *Casuarinaceae* field trials: effect of inoculation with *Frankia *on plant height.

Species	Treatments	Plant height (cm)	References
*Casuarina equisetifolia* (2-year-old field plantation)	Control	64.80	Karthikeyan et al., [50]
	*Frankia *	94.60	
*C. cunninghamiana* (6–8-year-old field plantation )	Control	580	Bulloch, [73]
	*Frankia *	680	
*C. glauca* (6–8-year-old field plantation )	Control	780	
	*Frankia *	890	
*C. obesa* (6–8-year-old field plantation )	Control	500	
	*Frankia *	550	
*Allocasuarina verticillata *	Control	580	
	*Frankia *	700	

**Table 2:** *Casuarinaceae* fields trials: effect of inoculation with *Frankia *on plants biomass and N content.

Species	Treatments	Dry weight (g)	Total N (g)/plant	References
*Casuarina equisetifolia* (3-year-old field plantation )	Control	1000	15.57	Sougoufara et al., [39]
	*Frankia *	1356	23. 35	
*Casuarina equisetifolia* (2-year-old field plantation )	Control	300	1.02	Karthikeyan et al., [50]
	*Frankia *	680	3.8	
*Alnus crispa* (1.5-year-old field plantation )	Control	111.8	3.5	Lefrançois et al., [79]
	*Frankia *	154.1	4.8	
